# The Effect of International Travel Arrivals on the New HIV Infections in 15–49 Years Aged Group Among 109 Countries or Territories From 2000 to 2018

**DOI:** 10.3389/fpubh.2022.833551

**Published:** 2022-02-16

**Authors:** Min Du, Jie Yuan, Wenzhan Jing, Min Liu, Jue Liu

**Affiliations:** Department of Epidemiology and Biostatistics, School of Public Health, Peking University, Beijing, China

**Keywords:** HIV, AIDS, travel, risk, 15–49 years

## Abstract

**Objective:**

The prevalent international travel may have an impact on new HIV infections, but related studies were lacking. We aimed to explore the association between international travel arrivals and new HIV infections in 15–49 years aged group from 2000 to 2018, to make tailored implications for HIV prevention.

**Methods:**

We obtained the data of new HIV infections from the Joint United Nations Programme on HIV/AIDS and international travel arrivals from the World Bank. Correlation analysis was used to explore the relation briefly. Log-linear models were built to analyze the association between international travel arrivals and new HIV infections.

**Results:**

International travel arrivals were positively correlated with new HIV infections (correlation coefficients: 0.916, *p* < 0.001). After controlling population density, the median age of the total population (years), socio-demographic index (SDI), travel-related mandatory HIV testing, HIV-related restrictions, and antiretroviral therapy coverage, there were 6.61% (95% CI: 5.73, 7.50; *p* < 0.001) percentage changes in new HIV infections of 15–49 years aged group associated with a 1 million increase in international travel arrivals.

**Conclusions:**

Higher international travel arrivals were correlated with new HIV infections in 15–49 years aged group. Therefore, multipronged structural and effective strategies and management should be implemented and strengthened.

## Introduction

HIV is a devastating infection, which weakens the body's ability to fight other infections by attacking the body's immune system, specifically the white blood cells called CD4 cells (1). Due to the impaired immune system, people living with HIV (PLHIV) will become more susceptible to other severe illnesses, such as tuberculosis and bacterial infections (2–7). HIV has been the most severe and widespread infectious disease pandemic in the world. At the end of 2019, WHO reported that an estimated 38 million PLHIV worldwide, an estimated annual number of new HIV infections was 1.7 million, and 770,000 people died of HIV-related illnesses worldwide in 2018 (8). WHO proposed an ambitious strategy for ending the HIV pandemic by 2030 (9). For this strategy to succeed, each HIV-afflicted country needs to achieve three goals, i.e., 95% of HIV-positive people knowing their status, 95% of people who know their HIV-positive status on treatment; and 95% of those on treatment virally suppressed. Despite a global downward trend of HIV incidence globally, several countries or regions are experiencing sharp increases in new infections and struggling to expand treatment (10), such as Russia, Ukraine, Southern and Eastern Africa, and some middle- and upper-income countries across North and South America, South and Southeast Asia (11). The European Center for Disease Prevention and Control underlined that one of five PLHIV was unaware of their status and only 43% of PLHIV have viral loads low enough to prevent transmission in Europe and central Asia (12). It is estimated that there were 7.1 million PLHIV did not know that they have HIV in 2018 globally (1). Therefore, some regions are not on track to meet the target for ending HIV, which is far lower than the final target (10).

In recent years, influenced social-cultural factors on HIV transmission grew out of a global concern, such as social inequalities (13), under-regulated clinics (14), child marriage, poverty (15), conflict (16), funding (10), immigrants (17), discrimination (18), and international travel (19–24). According to the World Tourism Organization, the number of international journeys exceeded 1.32 billion in 2017 and continued to grow (25). The total number of outbound passengers increased from 0.87 billion to 1.58 billion between 2010 and 2019 (26). Meanwhile, travel-related infectious diseases have been concerned. From 2014 to 2018, the incidence rate of post-travel infections was 14.20 per million, and 7.60% of them were blood/sexually transmitted diseases in China (27). French medical students who participated in an internship abroad during the summer months reported that acquisition rates of Gardnerella vaginalis and Atopobium vaginae were 12.9 and 13.9% in 2018 and 2019, respectively (28). Reports on a specific number of HIV infections among international travelers were lacking. One study reported that there were 38% primary HIV infections among 84 HIV-negative travelers (29). Geographically dispersed transmission of HIV infection was caused by the mobility of populations (30). In addition, international HIV-positive travelers' casual sexual activity with new partners and sex tourists' risk behaviors, such as condomless sex with multiple partners, may exacerbate the new HIV infections (31–34). International travel could connect areas of low HIV prevalence and areas of high HIV prevalence, especially the movement of high-risk individuals with unprotected anal intercourse and other risk behaviors (34).

Historically, travel is known to be associated with amplified risk of acquisition and transmission of infectious diseases, but studies that investigated the effect of international travel arrivals on the new HIV infections were scarce. This study is aimed to test whether international travel arrivals are correlated with the new HIV infections to make tailored implications for HIV prevention.

## Methods

### Study Design

This study was designed to evaluate the ecological correlation between international travel arrivals and new HIV infections in 15–49 years aged group over a 19-year period (2000–2018).

### Dependent and Explanatory Variables

The estimated data of new HIV infections in 15–49 years aged group, available from the Joint United Nations Programme on HIV/AIDS (UNAIDS, https://aidsinfo.unaids.org/), were used for the analysis in this paper. UNAIDS estimates the weighted average of new HIV infections (the Spectrum software) which refers to the annual number of new HIV infections among uninfected populations expressed per 1,000 uninfected population in the year.

The data of international travel arrivals, available from the World Bank (https://data.worldbank.org/?name_desc=false), were sourced from the World Tourism Organization, Yearbook of Tourism Statistics Compendium of Tourism Statistics, and data files. International travel arrivals are defined as the international inbound tourists (overnight visitors) who travel to a country other than that in which they have their usual residence, but outside their usual environment, for a period not exceeding 12 months and whose main purpose in visiting is other than an activity remunerated from within the country visited. The data on inbound tourists refer to the number of arrivals, not to the number of people who are traveling. Thus, a person who makes several trips to a country during a given period is counted each time as a new arrival.

### Control Variables

In our study, we included population density (persons per square km), the median age of the total population (years), socio-demographic index (SDI) values, antiretroviral therapy coverage (% of PLHIV), travel-related mandatory HIV testing, and HIV-related restrictions on entry, stay, and residence. Data of population density and the median age of the total population were available from United Nations (https://population.un.org/wpp/Download/Standard/Population/). The SDI developed by the Global Burden of Disease (GBD) study researchers was a composite indicator of total fertility rate under age 25 years, years of education for those ages 15 and older, and lag distributed income per capita (http://ghdx.healthdata.org/record/ihme-data/gbd-2019-socio-demographic-index-sdi-1950-2019). Data on antiretroviral therapy coverage, travel-related mandatory HIV testing, and HIV-related restrictions on entry, stay, and residence were available from UNAIDS (https://aidsinfo.unaids.org/). Travel-related mandatory HIV testing and HIV-related restrictions on entry, stay, and residence included four groups: (1) no restrictions; (2) require HIV testing or disclosure for some permits; (3) prohibit short- and/or long-stay and require HIV testing or disclosure for some permits; (4) deport, prohibit short and/ or long-stay, and require HIV testing or disclosure for some permits.

### Statistical Analysis

In the present study, we used the original data sourced from the UNAIDS or World Bank to calculated the total number of new HIV infections in 15–49 years aged group or international travel arrivals from 2000 to 2018. We used estimated annual percentage changes (EAPCs) to measure the trend of new HIV infections or international travel arrivals over a specified time interval. A regression line was fitted to the natural logarithm of the number of new HIV infections or international travel arrivals, that is, y = α + βx + ε, where y = ln (new HIV infections or international travel arrivals) and x = calendar year. EAPC (95% CIs) was calculated as 100 × (e^β^ – 1) to measure the temporal trend of new HIV infections or international travel arrivals in our study. If the EAPC estimation and its 95% CIs were both > 0 (or both <0), the new HIV infections or international travel arrivals had an increasing trend (or a decreasing trend).

Correlation analysis was used to analyze the relation between international travel arrivals and new HIV infections briefly. Then, we built log-linear models (GLM) to analyze the association in depth. In order to examine the robustness of the model, first, the basic model A was constructed, i.e., new HIV infections, international travel arrivals, population density, the median age of the total population (years), SDI. Second, based on model A, travel-related mandatory HIV testing and HIV-related restrictions on entry, stay, and residence was added in the model B. Third, model C was a full model based on model B by adding antiretroviral therapy coverage. All analyses were performed using R software (version 3.6.0) with the “mgcv” package (version 1.8–28). The results were expressed as percentage changes and 95% CIs in new HIV infections correlated with a 1 million increase in international travel arrivals. All tests were two-sided, and a value *p* < 0.05 was considered as statistical significance.

## Results

**Figure 1:**
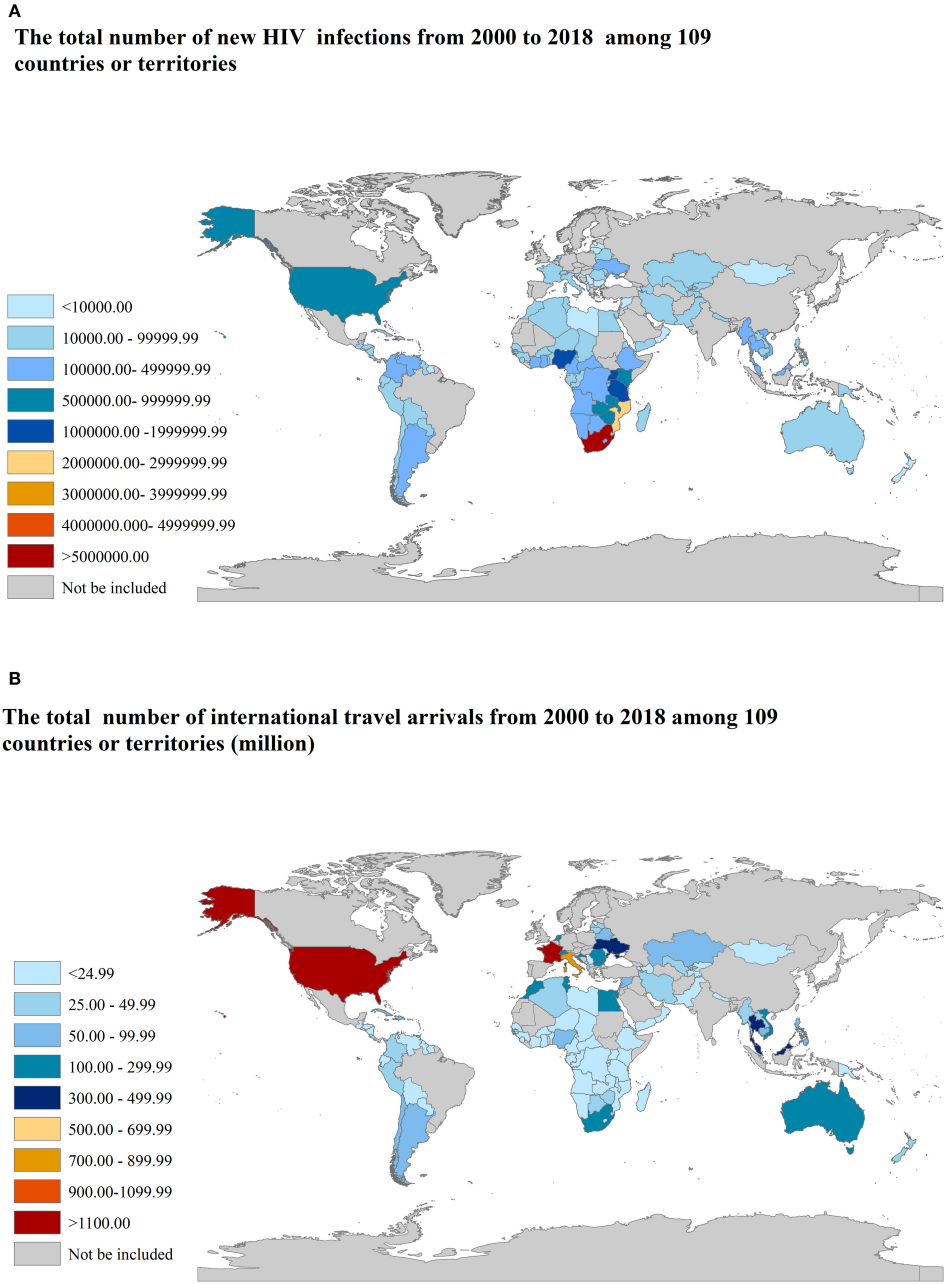
The total number of new HIV infections and international travel arrivals from 2000 to 2018 among 109 countries or territories. **(A)** new HIV infections; **(B)** international travel arrivals.

From 2000 to 2018, there were 109 countries or territories with at least one annual record on new HIV infections in 15–49 years aged group and international travel arrivals simultaneously, as shown in Figure 1. A total number of 24,226,800 new HIV infections in 15–49 years aged group and 8082.92 million international travel arrivals had been documented. The top three countries or territories with the highest total number of new HIV infections are South Africa, Mozambique, and Tanzania, as shown in Figure 1A. The top three countries or territories with the highest total number of international travel arrivals are France, the United States, and Italy, as shown in Figure 1B.

**Figure 2:**
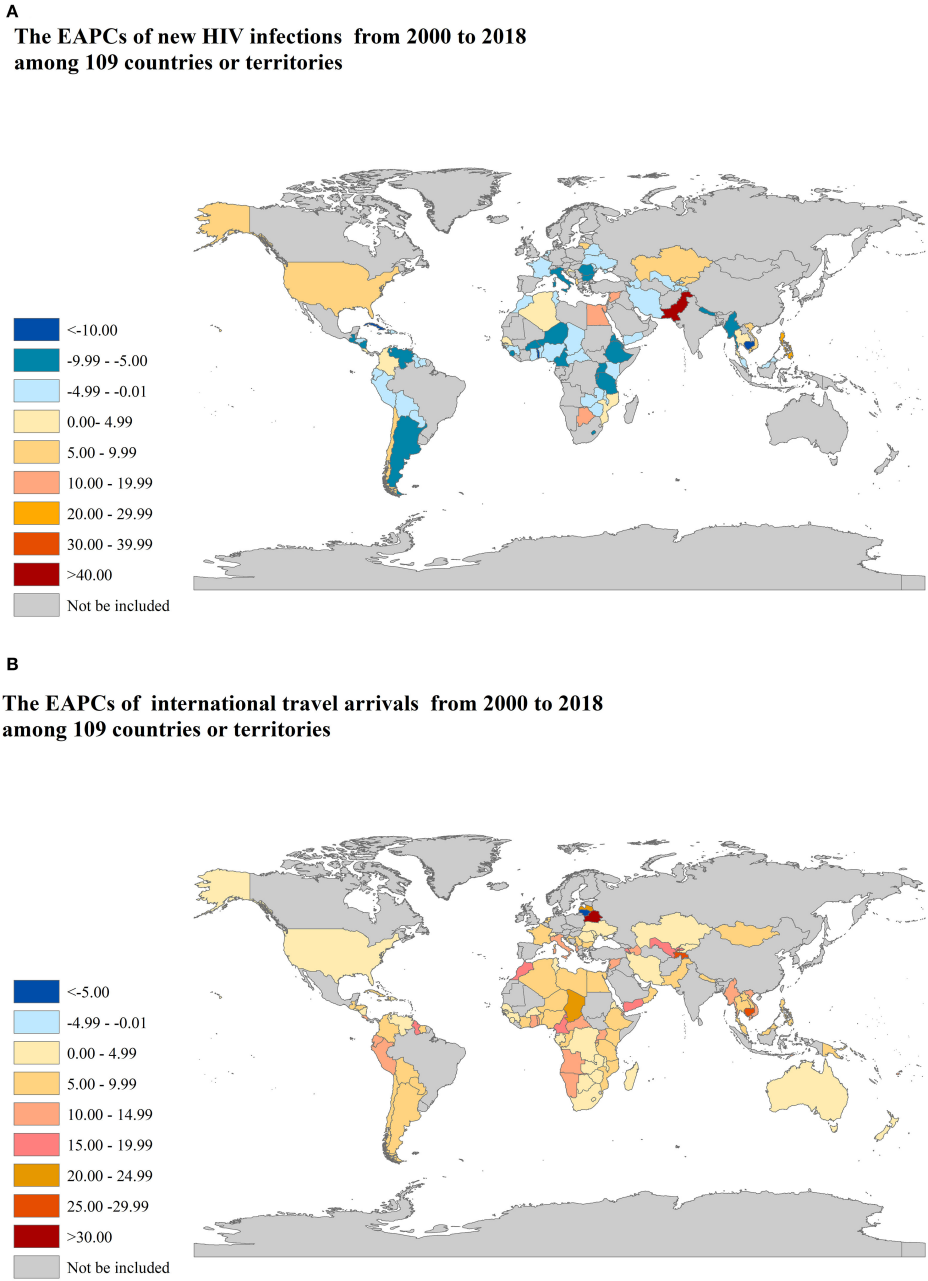
The EAPCs of new HIV infections and international travel arrivals from 2000 to 2018 among 109 countries or territories. EAPCs: estimated annual percentage changes. **(A)** new HIV infections; **(B)** international travel arrivals.

There are 27 countries or territories that have an increasing trend of the number of new HIV infections from 2000 to 2018, most of which are in Oman, followed by Peru, Syria, and Lithuania, etc., as shown in Figure 2A. There are 101 countries or territories that have an increasing trend of the number of international travel arrivals from 2000 to 2018, most of which are in Belarus, followed by Cote d'Ivoire, Central African Republic, and Tajikistan etc., as shown in Figure 2B. All results are shown in Supplementary Table S1.

International travel arrivals were positively correlated with new HIV infections (Correlation coefficients: 0.916, *p* < 0.001). Models A–C were established by GLM models. In model A, with controlling for population density, the median age of the total population and SDI, international travel arrivals were correlated with new HIV infections (*p* < 0.001). In model B, travel-related mandatory HIV testing and HIV-related restrictions on entry, stay, and residence was added, the correlation between international travel arrivals and new HIV infections still remained stable (*p* < 0.001). After controlling for all the confounders, there were 6.61% changes in new HIV infections of 15–49 years aged group correlated with a 1 million increase in international travel arrivals (*p* < 0.001). All results are shown in Table 1.

**Table 1:** The effect of international travel arrivals on the new HIV infections in 15–49 years aged group among 109 countries or territories from 2000 to 2018.

**Model A**			**Model B**			**Model C**		
**β**	**95% CI**	***P***	**β**	**95% CI**	***P***	**β**	**95% CI**	***P***
6.57%	5.82–7.32	<0.0001	6.63%	5.76–7.51	<0.0001	6.61%	5.73–7.50	<0.0001

*Model A adjusted population density, median age of the total population, SDI; travel-related mandatory HIV testing and HIV-related restrictions on entry, stay, and residence were added in Model B based on model A. All covariates were adjusted in Model C, such as population density, median age of the total population, SDI, antiretroviral therapy coverage, travel-related mandatory HIV testing, and HIV-related restrictions on entry, stay, and residence*.

## Discussion

To the best of our knowledge, our study is the first of its kind to suggest that international travel arrivals can be a substantial barrier to reduce the new HIV infections in 15–49 years aged groups. Our findings revealed that international travel arrivals were positively correlated with new HIV infections in 15–49 years aged group. For every 1 million increment in international travel arrivals, new HIV infections were increased by 6.61% in 15–49 years aged group. Although our study just correlated the number of new HIV diagnosis to the number of travels, which may not be directly related, the results still prompted that massive concerted and coordinated action need to be taken to reduce the new HIV infections.

Historically, travel is known to be associated with amplified risk of acquisition and transmission of infectious diseases, such as arboviruses (malaria, dengue, Zika, etc.), human respiratory syncytial virus, and histoplasmosis (35–39). Researchers found that there had high proportions of STI among travelers. Zöldi et al. investigated all Finnish international travel-related cases from 1995 to 2015 and reported that there were 2,304 travel-related STIs (26%) among 8,824 STIs (40). The attack rate was 0.76 per 100,000 travelers in 2015 for syphilis or gonorrhea among people returning from European countries (40). Wu et al. reported the incidence that post-travel illnesses was 14.20 per million with 1.07% blood/sexually transmitted diseases mainly imported from Southeast Asia to Yunnan and from Mongolia and Russia to Inner Mongolia (27). It can be seen that STI was transmitted by travelers internationally, crucially, limited studies reported the specific situation of travel-related HIV. One study reported that the prevalence of primary HIV infections was 38% among 84 HIV-negative travelers (29). Kutsuna et al. found that there were 7 travel-related HIV cases in Japan from 2017 to 2019 (24). Moreover, Wu et al. found that the HIV infection displayed a rapid increase among travelers from 2014 to 2018 in China (27). Therefore, it is necessary to explore the relationship between HIV infection and international travel.

Our study found that for every 1 million increment in international travel arrivals, new HIV infections increased by 6.61% in 15–49 years aged group. In fact, unlikely other diseases, such as malaria and yellow fever, which could be rapidly diagnosed because of their clinical manifestations or comprehensive testing policies, rapid detection is hard for HIV. Therefore, the correlation between international travel arrivals and new HIV infections may rely on specific individual-based behaviors. For example, countries with a high number of incoming travelers with different purposes may have different HIV risks, such as the number of sex tourists or sex workers and injection drugs' users. Sex tourists are a source of many international sexual health issues, due to high-risk behaviors, such as condomless sex with multiple partners or sexualized drug use while abroad (33). Of 1,189 men who have sex with men, 62 (5%) men were identified as sex tourists, of them, 20 (32%) traveled primarily to purchase sex and the remainder purchased sex while traveling for another purpose (34). MSM and MSM sex tourists were more likely to have risky sexual behaviors and travel to locations with a higher HIV prevalence (34). Of 290 HIV-positive people, 133 (45.9%) indicated that they had traveled internationally in the 5 years before the survey, 31 (23.3%) of them reported that they had casual sexual activity with new partners while traveling, and only 18 (58.1%) of them reported always using a condom (31). Sex transactions will increase the transmission of the virus, even establish a connection between areas of low HIV prevalence and areas of high HIV prevalence (34). Geographically dispersed transmission of HIV infection caused by the mobility of populations was a barrier, making it difficult to identify sex and drug partners who might be infected (30). Meanwhile, the local sex industry may be the other possible reason for the positive association between international travel arrivals and new HIV infections. The proportion of adolescent girls aged 10–19 years who sell sex is relatively high with an estimated 20% of the female sex-worker population in Ukraine (41).

The International AIDS Society recommends that global treatment efforts should be complemented with stronger investments in primary prevention, such as the development of a preventive vaccine (42). Currently, the HIV vaccine is undergoing late-stage clinical activity with three vaccine efficacy trials underway in southern Africa (42). However, it is difficult for people to use the HIV vaccine before traveling currently. In fact, some few countries had mandatory HIV testing for travelers (https://aidsinfo.unaids.org/), but international health authorities and societies highlighted that HIV testing must always respect personal choice and adhere to ethical and human rights principles and do not recommend mandatory, compulsory, or coerced HIV testing of individuals on public health grounds. Considering the above information, these suggestions were mentioned to control HIV transmission: (1) for travelers, it is best to seek pre-travel advice. Previous studies reported that only 35% of travelers received pre-travel advice (43). One-fourth of participants had a poor level of knowledge about the most common infectious diseases in the destination country (44). Therefore, people should learn about the local common infectious diseases, such as HIV, prevalence before international travel. It is better to avoid the risk behavior of HIV, such as unprotected sex with a new partner. (2) For sex workers, protected sex is essential whether regular clients or one-time clients. It is necessary to maintain routine health examinations and corresponding drug prevention and treatment for sex workers, such as mobile/migrant sex workers, especially for HIV-positive sex workers. (3) For health service providers, comprehensive sexual education, access to condoms, lubricant, and safe and confidential testing for HIV, etc., should be provided in public. There were only 31.2% of hospitals that provided pre-travel consulting, 72.0% of hospitals accepted travel-related patients out-of-hours in Japan (45). Additionally, there should be a pre-travel assessment for travelers, such as patient's medical history and immunization status, a detailed itinerary of the trip (such as activities, types of accommodation, and destination), and an assessment of the potential risks of possible exposure to HIV (46). A web-based self-reporting method for monitoring international passengers returning from an area of emerging infection is effective (47, 48). Health services providers should consider the situation of sex workers especially for mobile/migrant sex workers who may be linked to the higher burden of HIV through disrupted social networks and supports, reduced control over working conditions, and increased risks for violence, disruptions in antiretroviral therapy (ART) (49, 50). Therefore, efficient medical services, destigmatization, and elimination of sexual violence should be taken (51). Shannon et al. estimated that elimination of sexual violence alone could avert 17% of HIV infections in Kenya and 20% in Canada in the next decade (51). In Kenya, scaling up of access to antiretroviral therapy among female sex workers and their clients could avert 34% of infections and even modest coverage of sex worker led outreach could avert 20% of infections in the next decade (51). In short, to control the impact of international travel on the HIV epidemic, travelers, sex workers, and health service providers all need to do their best to implement control multipronged structural interventions.

There were some limitations in our study. Firstly, our study used the open dataset to explore the correlation between international travel arrivals and new HIV infections, so we may not cover all regions or countries in the world-based raw database. Secondly, new HIV infection was estimated by UNAIDS, which may have few differences compared with the specific epidemic situation of some regions or countries. Thirdly, because travel-related HIV testing is mandatory in very few countries, diagnosis for new HIV infection due to travels is difficult. Although we had controlled this factor, the specific impact of mandatory HIV testing for HIV new infections could not be measurable. Similarly, the residual confounding factors, such as travel's characteristics and behaviors, were not controlled due to the inaccessibility of data, these factors may affect our estimation. Last but not least, our results did not prove a causal association because our study used an ecological design. Moreover, causal inference and verification should be carried out through population cohort studies or randomized controlled trial studies in the future.

In conclusion, higher international travel was linked with increased new HIV infections. Although there was only an ecological correlation between international travel and new HIV infections, comprehensive control and preventive measures from various perspectives, such as travelers, sex workers, and health services providers, are crucial.

## Data Availability Statement

All data are from the public, open access repository (The estimated data of new HIV infections in 15–49 years aged group, available from the Joint United Nations Programme on HIV/AIDS UNAIDS, https://aidsinfo.unaids.org/; the data of international travel arrivals, available from World Bank, https://data.worldbank.org/?name_desc=false; Data of population density and median age of the total population was available from United Nations, https://population.un.org/wpp/Download/Standard/Population/; The SDI developed by the Global Burden of Disease study researchers (GBD), http://ghdx.healthdata.org/record/ihme-data/gbd-2019-socio-demographic-index-sdi-1950-2019; Data of antiretroviral therapy coverage, travel-related mandatory HIV testing and HIV-related restrictions on entry, stay and residence was available from UNAIDS, https://aidsinfo.unaids.org/).

## Ethics Statement

Ethical review and approval was not required for the study on human participants in accordance with the local legislation and institutional requirements. Written informed consent from the participants' legal guardian/next of kin was not required to participate in this study in accordance with the national legislation and the institutional requirements.

## Author Contributions

Conceptualization was done by MD, JL, and ML conceptualized and participated in writing—review and editing. Formal analysis was done by MD. MD, JY, and WJ participated in methodology. MD participated in writing—original draft. JL contributed to funding acquisition and supervision. All the authors have made substantial contributions to the conception, design of the work or the acquisition, analysis, or interpretation of data for the work. They have participated in drafting the manuscript and approval of the version to be published.

## Funding

This study was supported by the National Natural Science Foundation of China (grant numbers 72122001 and 71934002), the National Key Research and Development Project of China (grant numbers 2021ZD0114101, 2021ZD0114104, and 2021ZD0114105), and the National Statistical Science Research Project (grant number 2021LY038). The funding body had no role in the design or conduct of the study; the collection, management, analysis, or interpretation of the data; the preparation, review, or approval of the manuscript; or the decision to submit the manuscript for publication. We appreciate the works by UNAIDS, World Bank, United Nations, GBD.

## Conflict of Interest

The authors declare that the research was conducted in the absence of any commercial or financial relationships that could be construed as a potential conflict of interest.

## Publisher's Note

All claims expressed in this article are solely those of the authors and do not necessarily represent those of their affiliated organizations, or those of the publisher, the editors and the reviewers. Any product that may be evaluated in this article, or claim that may be made by its manufacturer, is not guaranteed or endorsed by the publisher.
