# Supplementary material for: The Effect of International Travel Arrivals on the New HIV Infections in 15–49 Years Aged Group Among 109 Countries or Territories From 2000 to 2018
Source: Front Public Health. 2022 Feb 16;10:833551. doi: 10.3389/fpubh.2022.833551 (PMC8888525; doi:10.3389/fpubh.2022.833551)
Supplement: Supplementary file 1 [file Table_1.DOCX]

**Supplemental Table 1 The total number and estimated annual percentage changes (EAPCs) of new HIV infections and international travel arrivals from 2000 to 2018 among 109 countries or territories**

| **Countries or territories** | **New HIV infections** | | **International travel arrivals (million)** | |
| --- | --- | --- | --- | --- |
|  | **Total** | **EAPC (95% CI)** | **Total** | **EAPC (95% CI)** |
| Total | 24226800 | -2.87 (-7.47, 1.97) | 8082.92 | 3.71 (3.17, 4.25) |
| Albania | 1400 | 7.44 (5.97, 8.92) | 35.87 | 14.69 (12.07, 17.38) |
| Algeria | 21100 | 2.68 (1.88, 3.48) | 34.65 | 6.03 (4.40, 7.68) |
| Angola | 329000 | 0.22 (-0.14, 0.57) | 5.84 | 11.34 (6.58, 16.32) |
| Argentina | 101500 | -6.83 (-8.84, -4.77) | 97.18 | 6.25 (5.05, 7.46) |
| Armenia | 7100 | - | 13.44 | 17.84 (14.80, 20.96) |
| Australia | 19000 | -0.36 (-0.79, 0.08) | 117.12 | 3.44 (2.76, 4.13) |
| Azerbaijan | 17200 | -4.98 (-6.59, -3.34) | 24.66 | 10.11 (8.09, 12.16) |
| Barbados | 2600 | 5.95 (4.34, 7.59) | 10.64 | 1.00 (0.38, 1.62) |
| Belarus | 28600 | -2.12 (-2.28, -1.96) | 56.65 | 42.62 (29.79, 56.73) |
| Benin | 66500 | -0.66 (-1.29, -0.03) | 3.72 | 6.23 (4.48, 8.01) |
| Bolivia | 19500 | -3.99 (-4.55, -3.42) | 12.64 | 7.23 (6.61, 7.85) |
| Botswana | 218800 | 10.51 (8.15, 12.92) | 26.78 | 1.92 (0.51, 3.34) |
| Bulgaria | 5900 | -5.72 (-5.90, -5.54) | 111.38 | 6.04 (5.33, 6.75) |
| Burkina Faso | 64600 | -5.44 (-5.79, -5.09) | 3.89 | 0.05 (-2.53, 2.69) |
| Burundi | 45600 | -6.25 (-6.87, -5.63) | 2.83 | 9.36 (4.87, 14.04) |
| Cambodia | 52800 | -11.59 (-12.88, -10.28) | 52.32 | 27.67 (20.11, 35.70) |
| Cameroon | 270000 | -5.04 (-5.66, -4.43) | 8.48 | 15.13 (13.58, 16.69) |
| Cape Verde | 3400 | -3.58 (-5.37, -1.77) | 6.70 | 8.85 (7.52, 10.19) |
| Central African Republic | 126400 | -3.99 (-4.34, -3.64) | 0.84 | 11.15 (10.27, 12.02) |
| Chad | 49900 | -2.76 (-3.24, -2.27) | 1.01 | 22.24 (17.08, 27.63) |
| Chile | 56500 | 6.13 (4.93, 7.34) | 59.57 | 6.35 (3.08, 9.73) |
| Colombia | 175700 | 1.30 (0.56, 2.05) | 37.30 | 8.18 (7.16, 9.21) |
| Comoros | 1900 | - | 0.42 | 11.54 (8.84, 14.31) |
| Congo (Brazzaville) | 70700 | 2.68 (2.39, 2.97) | 2.27 | 1.56 (-0.82, 4.00) |
| Costa Rica | 17000 | 3.72 (1.80, 5.67) | 38.57 | 13.99 (6.06, 22.51) |
| Cote d’Ivoire | 187000 | - | 9.07 | 6.07 (5.36, 6.78) |
| Croatia | 1900 | 3.83 (2.22, 5.46) | 187.00 | 5.19 (4.63, 5.76) |
| Cuba | 30600 | -11.68 (-15.05, -8.18) | 50.85 | 5.57 (4.80, 6.35) |
| Djibouti | 7900 | -5.44 (-6.57, -4.30) | 0.57 | 10.52 (8.72, 12.35) |
| Dominican Republic | 70400 | -3.42 (-3.57, -3.27) | 82.15 | 4.53 (4.06, 5.01) |
| Congo Demographic Republic | 411000 | -0.18 (-0.51, 0.15) | 2.19 | 14.87 (8.87, 21.21) |
| Ecuador | 45300 | 12.76 (10.87, 14.68) | 22.25 | 7.00 (6.18, 7.83) |
| Egypt | 25400 | -5.69 (-6.37, -5.00) | 166.93 | 3.12 (0.36, 5.95) |
| El Salvador | 31900 | -5.45 (-7.40, -3.46) | 22.51 | 4.04 (2.89, 5.21) |
| Eritrea | 14000 | -3.34 (-4.63, -2.03) | 1.68 | 2.98 (0.94, 5.07) |
| ESwatini | 189800 | -5.70 (-5.94, -5.45) | 14.05 | 6.52 (3.76, 9.36) |
| Ethiopia | 380000 | 2.09 (0.39, 3.82) | 9.09 | 12.46 (11.29, 13.65) |
| Fiji | 2100 | -1.26 (-2.19, -0.32) | 11.32 | 5.12 (4.30, 5.94) |
| France | 95500 | -5.67 (-6.68, -4.66) | 1342.22 | 0.72 (0.46, 0.98) |
| Gabon | 14200 | -0.53 (-1.19, 0.13) | 1.27 | 11.86 (9.14, 14.65) |
| Ghana | 266000 | -6.33 (-7.32, -5.32) | 10.82 | 6.21 (4.61, 7.83) |
| Guatemala | 17600 | -2.86 (-3.09, -2.62) | 16.60 | 2.76 (0.03, 5.56) |
| Guinea | 90000 | -2.64 (-3.33, -1.95) | 0.87 | 3.24 (-2.10, 8.87) |
| Guinea-Bissau | 28600 | -4.98 (-6.59, -3.34) | 0.33 | 15.98 (11.25, 20.91) |
| Guyana | 13000 | -2.97 (-3.42, -2.53) | 3.03 | 5.95 (5.26, 6.65) |
| Haiti | 130900 | -1.98 (-3.33, -0.61) | 5.62 | 9.66 (6.70, 12.69) |
| Honduras | 19600 | -2.05 (-2.23, -1.86) | 13.64 | 3.45 (2.42, 4.50) |
| Iran | 50600 | -5.04 (-5.91, -4.16) | 48.57 | 11.51 (8.71, 14.39) |
| Italy | 81900 | -1.60 (-2.23, -0.97) | 859.73 | 2.31 (1.75, 2.88) |
| Jamaica | 32500 | 7.96 (7.20, 8.73) | 34.17 | 3.76 (3.39, 4.13) |
| Kazakhstan | 34700 | -3.25 (-3.70, -2.81) | 96.40 | 6.53 (4.82, 8.27) |
| Kenya | 961000 | 8.86 (5.51, 12.31) | 23.07 | 2.47 (0.54, 4.43) |
| Kyrgyzstan | 12900 | - | 4.65 | 9.72 (7.13, 12.37) |
| Laos | 19000 | - | 31.98 | 20.63 (17.38, 23.97) |
| Latvia | 9500 | 5.50 (3.77, 7.25) | 26.70 | 6.26 (4.40, 8.16) |
| Lebanon | 3000 | -5.01 (-5.49, -4.53) | 26.07 | 4.39 (2.72, 6.08) |
| Lesotho | 311700 | - | 10.46 | 9.04 (6.29, 11.86) |
| Libya | 2000 | 5.62 (2.67, 8.65) | 0.62 | -8.00 (-21.06, 7.22) |
| Lithuania | 7700 | 14.27 (12.90, 15.66) | 34.66 | 3.57 (2.16, 5.00) |
| Madagascar | 39300 | -2.85 (-3.23, -2.47) | 4.41 | 3.43 (0.11, 6.87) |
| Malawi | 782000 | -0.75 (-1.02, -0.47) | 12.30 | 6.70 (4.82, 8.61) |
| Malaysia | 102400 | -1.25 (-1.95, -0.55) | 394.95 | 5.46 (4.14, 6.80) |
| Mauritius | 20400 | -1.99 (-2.95, -1.02) | 17.74 | 4.20 (3.75, 4.66) |
| Moldova | 16800 | - | 1.34 | 6.26 (4.25, 8.30) |
| Mongolia | 1900 | - | 7.12 | 5.23 (3.19, 7.31) |
| Montenegro | 1700 | -1.51 (-1.84, -1.18) | 17.58 | 18.20 (13.40, 23.20) |
| Morocco | 21400 | 0.71 (0.21, 1.22) | 151.86 | 6.25 (5.48, 7.03) |
| Mozambique | 2110000 | -5.08 (-5.39, -4.76) | 23.10 | 11.41 (8.34, 14.58) |
| Myanmar | 314800 | -3.59 (-4.00, -3.17) | 28.51 | 13.89 (10.74, 17.14) |
| Namibia | 157700 | -9.21 (-10.11, -8.30) | 17.89 | 5.16 (4.68, 5.65) |
| Nepal | 42600 | -2.88 (-4.64, -1.08) | 11.25 | 6.43 (4.69, 8.21) |
| Netherlands | 17500 | 2.09 (0.39, 3.82) | 227.82 | 3.64 (2.75, 4.54) |
| New Zealand | 3600 | -5.21 (-6.76, -3.63) | 48.51 | 3.37 (2.77, 3.98) |
| Nicaragua | 15000 | -7.49 (-9.12, -5.83) | 18.38 | 7.47 (6.56, 8.39) |
| Niger | 31500 | -0.52 (-0.84, -0.20) | 1.67 | 8.05 (6.60, 9.52) |
| Nigeria | 1403000 | - | 67.81 | 8.02 (4.99, 11.13) |
| Oman | 2800 | 49.71 (32.30, 69.42) | 21.64 | 6.67 (4.22, 9.18) |
| Pakistan | 95300 | -1.35 (-2.97, 0.30) | 9.95 | 6.84 (4.70, 9.03) |
| Papua New Guinea | 47200 | -3.44 (-4.65, -2.21) | 2.23 | 7.96 (6.06, 9.90) |
| Paraguay | 24800 | -0.98 (-1.26, -0.69) | 11.52 | 10.13 (8.27, 12.02) |
| Peru | 69100 | 22.68 (20.74, 24.66) | 44.43 | 9.59 (8.91, 10.28) |
| Philippines | 93000 | - | 70.98 | 8.01 (7.39, 8.63) |
| Romania | 19000 | -6.37 (-7.33, -5.40) | 144.93 | 4.49 (3.65, 5.34) |
| Rwanda | 100800 | -8.44 (-10.29, -6.55) | 8.00 | 14.99 (11.19, 18.91) |
| Senegal | 20500 | 3.63 (1.35, 5.97) | 12.62 | 3.69 (2.43, 4.98) |
| Serbia | 3100 | 0.13 (-0.16, 0.42) | 13.78 | 10.38 (9.39, 11.38) |
| Sierra Leone | 71200 | -7.41 (-10.46, -4.25) | 0.79 | 4.49 (1.77, 7.28) |
| Singapore | 7800 | -4.99 (-5.58, -4.39) | 174.87 | 5.95 (5.08, 6.82) |
| South Africa | 6770000 | - | 157.12 | 3.13 (2.32, 3.95) |
| Suriname | 9000 | -0.40 (-3.12, 2.40) | 3.10 | 9.79 (7.44, 12.19) |
| Switzerland | 11000 | - | 151.73 | 1.96 (1.45, 2.47) |
| Syria | 1200 | 14.59 (8.68, 20.82) | 50.05 | 11.92 (8.74, 15.19) |
| Tajikistan | 11700 | -0.71 (-1.02, -0.40) | 3.78 | 28.30 (19.27, 38.01) |
| Tanzania | 1414000 | -9.15 (-9.49, -8.82) | 15.78 | 6.31 (5.77, 6.85) |
| Thailand | 345400 | 1.04 (0.69, 1.38) | 366.09 | 8.38 (7.45, 9.31) |
| The Gambia | 30300 | 7.91 (4.52, 11.42) | 3.72 | 11.32 (8.08, 14.66) |
| Timor-Leste | 1800 | -3.93 (-4.31, -3.54) | 0.68 | 12.13 (7.44, 17.02) |
| Togo | 98700 | -12.90 (-15.48, -10.24) | 3.85 | 14.50 (12.19, 16.85) |
| Trinidad and Tobago | 12000 | 12.56 (9.81, 15.37) | 7.99 | -0.05 (-0.66, 0.56) |
| Tunisia | 11400 | -0.76 (-1.55, 0.03) | 122.68 | 1.62 (0.42, 2.85) |
| Uganda | 1121000 | -5.00 (-5.84, -4.15) | 14.56 | 12.93 (10.77, 15.12) |
| Ukraine | 330000 | -0.60 (-0.98, -0.22) | 317.31 | 2.26 (-0.94, 5.57) |
| United States | 691000 | 8.21 (6.89, 9.53) | 1147.07 | 3.71 (3.07, 4.36) |
| Uzbekistan | 44800 | -4.31 (-4.97, -3.65) | 25.54 | 17.16 (13.89, 20.53) |
| Venezuela | 131100 | -8.21 (-9.45, -6.97) | 11.55 | 2.42 (-0.29, 5.21) |
| Vietnam | 291300 | 6.31 (3.93, 8.74) | 111.46 | 10.88 (9.80, 11.98) |
| Yemen | 13000 | -0.25 (-0.44, -0.05) | 10.17 | 17.95 (8.99, 27.65) |
| Zambia | 896000 | -3.96 (-4.30, -3.62) | 14.79 | 4.90 (3.72, 6.10) |
| Zimbabwe | 984000 | -2.85 (-3.67, -2.02) | 40.06 | 0.54 (-0.61, 1.71) |
